# Modern contraceptive use among female refugee adolescents in northern Uganda: prevalence and associated factors

**DOI:** 10.1186/s12978-020-00921-y

**Published:** 2020-05-20

**Authors:** Ritah Bakesiima, Amanda Cleeve, Elin Larsson, James K. Tumwine, Grace Ndeezi, Kristina Gemzell Danielsson, Rose Chalo Nabirye, Jolly Beyeza Kashesya

**Affiliations:** 1 Department of Obstetrics and Gynaecology, School of Medicine, College of Health Sciences, Makerere University, Kampala, Uganda; 2 Department of Women’s and Children’s Health, Division of Obstetrics and Gynaecology, Karolinska Institutet, and Karolinska University Hospital, Stockholm, Sweden; 3 Department of Women’s and Children’s Health, Uppsala University, Uppsala, Sweden; 4 Department of Paediatrics and Child health, School of Medicine, College of Health Sciences, Makerere University, Kampala, Uganda; 5 Department of Nursing, School of Health Sciences, College of Health Sciences, Makerere University, Kampala, Uganda

**Keywords:** Modern contraceptives, Contraception, Refugees, Adolescents, Teenage pregnancies

## Abstract

**Background:**

Adolescent pregnancies are persistently high among refugees. The pregnancies have been attributed to low contraceptive use in this population. The aim of this study was to determine the prevalence and factors associated with modern contraceptive use among female refugee adolescents in northern Uganda.

**Methods:**

This was a cross sectional study using both descriptive and analytical techniques. The study was carried out in Palabek refugee settlement in Northern Uganda from May to July 2019. A total of 839 refugee adolescents who were sexually active or in-union were consecutively enrolled. Interviewer administered questionnaires were used for data collection.

**Results:**

Modern contraceptive prevalence was 8.7% (95% CI: 7.0 to 10.8). The injectable was the most commonly used modern contraceptive method [42.5% (95% CI: 31.5 to 54.3)], and most of the participants had used the contraceptives for 6 months or less (59.7%). Reasons for not using modern contraceptives included fear of side effects (39.3%), partner prohibition (16.4%), and the desire to become pregnant (7.0%). Participants who were married (OR = 0.11, 95% CI: 0.04 to 0.35, *p* < 0.001), cohabiting (OR = 0.43, 95% CI: 0.20 to 0.93, *p* = 0.032) or having an older partner (OR = 0.93, 95% CI: 0.86 to 0.99, *p* = 0.046) were less likely to use modern contraceptives.

**Conclusion:**

Modern contraceptive use among female refugee adolescents was very low, and few reported a desire to become pregnant, leaving them vulnerable to unplanned pregnancies. Least likely to use modern contraceptives were participants who were married/cohabiting and those having older partners implying a gender power imbalance in fertility decision making. There is an urgent need for innovations to address the gender and power imbalances within relationships, which could shape fertility decision-making and increase modern contraceptive use among refugee adolescents.

## Plain English summary

Adolescent pregnancies are persistently high globally, with higher rates reported among refugees and migrants. These expose the affected girls to the complications associated with teenage pregnancy and child birth, which are the leading cause of death among girls aged 15 to 19 years globally. The high rates of adolescent pregnancies have been attributed to low contraceptive use amongst the adolescents. However, there is scarce information on contraceptive use among refugee adolescents. A total of 839 female refugee adolescents were enrolled in order to determine the prevalence and factors associated with modern contraceptive use in this population in Palabek refugee settlement, Northern Uganda. It was found that modern contraceptives were used by less than a tenth of the participants, and yet the majority did not want to get pregnant. Since the participants were sexually active, their low use of modern contraceptives meant that they were vulnerable to unwanted or unplanned pregnancy and its associated complications. Some of the reasons for not using modern contraceptives included fear of side effects and partner prohibition. Adolescents who were married/cohabiting, and those who had older partner were less likely to use modern contraceptives. This could be explained by gender power imbalances which leave the adolescents with lower negotiating power compared with adult women, especially surrounding matters that concern their sexual and reproductive health. There is an urgent need for measures to improve adolescents’ access to high-quality sexual and reproductive healthcare in refugee settings, with more emphasis put on empowering them to make decisions about their own health including initiating a contraceptive method. Interventions should also aim at addressing fear of side effects and increasing knowledge on modern contraceptives.

## Introduction

Refugees are rapidly increasing worldwide with the number reaching 25.9 million [1]. Uganda is the third largest refugee hosting nation in the world, and the largest in Africa with over 1.3 million refugees from South Sudan, the Democratic Republic of Congo, and Burundi [2]. Over 61% of these refugees are below 18 years [3].

Refugees are a vulnerable group because of the conflicts, insecurity, and the violence and poverty they often face. Globally, women and children constitute over 80% of refugee populations [4]. Female refugee adolescents are especially vulnerable due to high risk of sexual violence, exploitation and abuse, and early or forced marriage [4, 5]. Adolescent pregnancies are reported to be higher among refugees or displaced persons than the non-displaced persons at 30 and 19% respectively [6].

Adolescent pregnancy can be life threatening because of the complications associated with pregnancy and child birth in this age group. Such complications include obstetric fistula, pregnancy induced hypertension, haemorrhagic syndrome, premature rupture of membranes and unsafe abortions among others [7–10]. These complications are the leading cause of death among girls aged 15 to 19 years globally [11].

In 2006, Uganda allowed contraceptive use among sexually active adolescents in order to curb teenage pregnancy rates and associated complications [12, 13]. However, many adolescents are still getting pregnant due to low contraceptive use [14, 15]. In Uganda, modern contraceptive use among married and unmarried sexually active female adolescents is 25.1% [16]. This persistently low use and high teenage pregnancy rate in Uganda has been attributed to a number of reasons such as unpredictable and irregular sexual activity, limited access to contraception, inadequate knowledge on contraceptives and fear of side effects [17, 18].

When it comes to refugee settings in Uganda, little is known about adolescents’ contraceptive use, and sexual and reproductive practices. The aim of this study was therefore to determine the prevalence and factors associated with modern contraceptive use among female refugee adolescents in Northern Uganda. This information is crucial for improving sexual and reproductive health outcomes among refugee adolescents.

## Materials and methods

### Study design and setting

This was a cross sectional study carried out in Palabek refugee settlement from May to July, 2019. Palabek refugee settlement is located in the northern region of Uganda in Lamwo district and has served as a refugee settlement to over 38,000 refugees from South Sudan, 86% of whom are women and children [4, 19]. The settlement is arranged in zones, which are further divided into blocks. Every block has a community meeting place where members usually meet when summoned by their leaders for any communication.

Palabek refugee settlement has four health centres all of which provide contraceptive services freely to the refugees and to the host community. The contraceptive services provided by the health centres include counselling, giving out of oral contraceptives and condoms, insertion and removal of intra-uterine devices (IUDs) and implants, and giving of injectable contraceptives. Both adolescents and adults receive similar care regarding counselling, privacy and provision of contraceptives. These services are available most of the time, with stock-outs occurring infrequently.

### Participants

Female refugee adolescents were consecutively enrolled into this study. Block leaders asked female refugees to converge in their community meeting places within the block. In the meeting place, adolescents were informed about the study, and those who were willing to participate were screened for eligibility. The inclusion criteria were female refugees aged 10 to 19 years, who were in any form of union or unmarried but reported having had sex in the past 3 months, were settled with in Palabek refugee settlement from May to July 2019, and consented to participate in the study. Adolescents were excluded if they were physically or mentally unable to adhere to study procedures like consenting and responding to the interview questions. All adolescents who were informed about the study and were eligible consented to participate in the study.

### Sample size calculation

The sample size was calculated using the Kish Leslie formula [20] for the prevalence objective, in which we assumed an expected prevalence of 50%, meant to give us the largest sample size, and an absolute error or precision of 0.05. We also factored in non-response of 10%. This gave a sample size of 424.

To determine the factors associated with modern contraceptive use, the sample size formula for comparing means in two proportions by Hulley [21] was used. We assumed an error of 0.05, power of 80%, that the proportion of adolescents who are currently married and are using modern contraceptives is 20.7% [16], while the proportion of unmarried sexually active adolescents using modern contraceptives is 40.3% [16]. We also used the proportion of adolescents who are currently married in Uganda as 5.6% [16]. This gave a sample size of 839. Since this sample size was larger than that obtained from the Kish Leslie formula, it was used as the overall sample size for the study.

### Data collection

Data was collected using a pretested interviewer administered questionnaire. The interviewers were two male and two female research assistants working as village health team members (It is important to note that the gender of the interviewers did not have any significant effect on the responses of the participants according to our post hoc analysis). The interviewers were trained in medical research ethics and given guidance on how to administer the questionnaires. Information obtained with the use of the questionnaire included social demographics, sexual and reproductive history, spousal information and knowledge and use of modern contraceptives. The interviews took place at the community meeting place, in a private space away from other participants where conversations could not be over heard. This was done immediately after informing them about the study, and obtaining their consent to participate. The primary outcome variable was modern contraceptive use while the secondary outcome variables were: types of modern contraceptives used and duration of use of modern contraceptives.

### Statistical analysis

Data was analysed using STATA version 14.0 (StataCorp. 2014. *Stata Statistical Software: Release 14*). All continuous variables were summarised as means and standard deviations if they were normally distributed, and as medians and ranges if skewed. Categorical variables were summarised as percentages and proportions. Modern contraceptive use was analysed as a categorical variable, with use of modern contraceptives coded as “1” and non-use as “0”. Prevalence of use was calculated as the percentage of female refugee adolescents currently using or had used within the past 3 months any modern contraceptives over the total number of participants in the study.

Factors associated with modern contraceptive use were assessed using the logistic regression model. Bivariate analysis was done by fitting a model for all the independent variables with the outcome. All variables that were plausible considering prior knowledge and those gave a *p*-value ≤0.2 at the bivariate analysis were considered for multivariate analysis. At the multivariate analysis, variables were considered statistically significant if they had a *p*-value less than 0.05. Two-way product terms were formed for the significant variables and were used to assess for interaction using the chunk test. Where necessary, confounding was assessed for and a variable was considered to be a confounder if it caused a greater than or equal to 10% change in the odds ratio of modern contraceptive use. Odds ratios were presented along with their 95% confidence intervals, and statistical significance reported at *p* < 0.05.

## Results

### Background characteristics of the study participants

A total of 839 female adolescents were consecutively enrolled into the study. The age range was 15 to 19 years, and the mean age was 18.3 years (SD = 0.83). As shown in Table 1, seven hundred and twenty-seven (86.7%) of the participants were adolescents aged 18 years and above and 544 (64.8%) were either married or cohabiting. Considering the sexual and reproductive history, 169 participants (20.1%) had their first sexual intercourse below 16 years, 591 (70.4%) had ever been pregnant, 432 (73.1%) of whom said that at least one of the pregnancies was unintended. Amongst those who had unintended pregnancies, 15 (3.5%) opted to have an abortion while the rest prepared to give birth.

**Table 1:** Background characteristics of study participants

Variable	Number (*N* = 839)	Percentage (%)
**Age**		
15–17	116	13.8
18–19	723	86.2
**Religion**		
Catholic	440	52.4
Anglican	193	23.0
Moslem	1	0.1
Adventist	66	7.9
Other (Pentecostal, Lutheran, EFC, AIC)	139	16.6
**Ethnicity**		
Acholi	659	78.6
Dinka	19	2.2
Nuer	18	2.1
Lotuho	46	5.5
Other (Bari, Shilluk, Luo)	97	11.6
**Education**		
None	81	9.7
Primary	595	70.9
Secondary	148	17.6
Tertiary	15	1.8
**Occupation**		
Self-employed / Employed	33	3.9
Unemployed	335	39.9
Peasant farmer	305	36.4
Student	166	19.8
**Marital status**		
Single	233	27.8
Cohabiting	381	44.4
Married	163	19.4
Separated/Divorced/Widowed	62	7.4
**Age at first sex**		
12 to 15	169	20.1
16 to 17	537	64.0
18 to 19	133	15.9
**Ever been pregnant**		
Yes	591	70.4
No	248	29.6
**Number of children alive (n-591)**		
0	31	5.3
1	275	46.5
2–4	285	48.2
**Partner’s age**^**a**^ (Median = 26, Range = 16 to 60)		
16 to 25	281	43.5
26 to 35	331	51.3
36–60	30	4.6
**Partner’s Education**		
None	13	2.0
Primary	190	29.4
Secondary	423	65.5
Tertiary	20	3.1
**Partner’s Occupation**		
Self-employed / Employed	193	29.9
Unemployed	267	41.3
Peasant farmer	129	20.0
Student	57	8.8

*AIC* African Initiated Church, *EFC* Evangelical Free Church

^a^Missing data

### Knowledge and accessibility of modern contraceptives among the study participants

A total of 758 participants (90.3%) had ever heard about modern contraceptives, 513 (67.7%) had heard about modern contraceptives from a health worker, 124 (16.4%) from family and friends, 83 (10.9%) from school and 38 (5%) from the media (newspaper, television, radio, drama and posters). A good number of participants (82.1%) knew at least two modern contraceptives and the commonly known type was the condom (70.3%).

Regarding accessibility of the modern contraceptives, almost all the participants (99.6%) mentioned that the health facility was the only source of modern contraceptives they knew. A total of 589 (70.2%) of the participants did not know a contraceptive source within 10 min’ walk from their homes.

### Modern contraceptive use among the study participants

The prevalence of modern contraceptive use among the 839 participants was 8.7% (95% CI: 7.0–10.8). Amongst the users, 31 (42.5%) were using injectable contraceptives and 40 (59.7%) had used the contraceptives for 6 months or less. The main reasons for not using modern contraceptives given by 301 (39.3%) of the non-users was fear of side effects (Table 2).

**Table 2:** Modern contraceptive use among refugee adolescents in Northern Uganda

Variable	Number	Percentage	95% Confidence Interval
**Use of modern contraceptives (*n*** **= 839)**			
Yes	73	8.7	7.0 to 10.8
No	766	91.3	89.2 to 93.0
**Method of modern contraceptive used (*n*** **= 73)**			
Condom	12	16.4	9.5 to 27.1
Oral contraceptive (pill)	4	5.5	2.0 to 14.0
Injectable contraceptive	31	42.5	31.5 to 54.3
Implant	26	35.6	25.3 to 47.5
Intra-Uterine Device (IUD)	0	0.0	
**Duration of use (*n*** **= 67)**			
6 months or less	40	59.7	47.3 to 71.0
7 to 12 months	18	26.9	17.4 to 39.0
More than 12 months	9	13.4	7.0 to 24.2
**Reasons for non – use (*n*** **= 766)**			
Infrequent sex	83	10.8	7.6 to 12.4
Cultural / Religious prohibitions	85	11.2	9.0 to 13.5
Partner prohibitions	126	16.4	14.0 to 19.2
Fear of side effects	301	39.3	35.8 to 42.8
Lack of knowledge	105	13.7	11.4 to 16.3
Use of traditional methods	12	1.6	0.8 to 3.5
Want to become pregnant	54	7.0	5.4 to 10.1

### Prevalence of modern contraceptive use by participants’ background characteristics

According to the participants’ background characteristics, participants who had attained up to secondary education had a higher modern contraceptive prevalence (10.1%) compared to their counterparts. Contraceptive use was slightly more common among those who had lived in the camp for more than 12 months (8.9%) compared to those who had lived in the camp for a shorter period of time (7.4%). Participants who had ever been pregnant had almost the same prevalence of modern contraceptive use (8.8%) as those who had never been pregnant (8.4%). Furthermore, participants whose partners were students had a higher modern contraceptive prevalence rate (19.3%) compared to those whose partners had other occupations, as shown in Table 3.

**Table 3:** Prevalence of modern contraceptive use by participants’ background characteristics

Variable	Contraceptive use No. (%)	Contraceptive Non-use No. (%)	95% Confidence Interval
**Age**			
15 to 17	10 (8.6)	106 (91.4)	4.7 to 15.3
18 to 19	63 (8.7)	660 (91.3)	6.9 to 11.0
**Religion**			
Catholic	38 (8.6)	402 (91.4)	6.3 to 11.7
Anglican	15 (7.8)	178 (92.2)	4.7 to 12.5
Adventist	6 (9.1)	60 (90.9)	4.1 to 18.9
Other (Pentecostal, Lutheran, EFC, AIC)	13 (8.0)	108 (90.0)	6.0 to 16.2
**Ethnicity**			
Acholi	65 (9.9)	594 (90.1)	7.8 to 12.4
Dinka	1 (5.6)	17 (94.4)	0.7 to 32.0
Nuer	1 (5.3)	18 (94.7)	0.7 to 30.6
Lotuho	3 (6.5)	43 (93.5)	2.1 to 18.6
Other (Bari, Shulluk, Luo)	3 (3.1)	94 (96.9)	1.0 to 9.2
**Education**			
None	4 (4.9)	77 (95.1)	1.9 to 12.5
Primary	53 (8.9)	542 (91.1)	6.9 to 11.5
Secondary	15 (10.1)	133 (89.9)	6.2 to 16.2
Tertiary	1 (6.7)	14 (93.3)	0.9 to 36.9
**Occupation**			
Self-employed/Employed	3 (9.1)	30 (90.9)	2.9 to 25.1
Unemployed	29 (8.7)	306 (91.3)	6.1 to 12.2
Peasant farmer	23 (7.5)	282 (92.5)	5.1 to 11.1
Student	18 (10.8)	148 (89.2)	6.1 to 16.6
**Marital status**			
Single	19 (8.2)	214 (91.8)	5.3 to 12.4
Cohabiting	42 (11.0)	339 (88.0)	8.2 to 14.6
Married	6 (3.7)	157 (96.3)	1.7 to 8.0
Separated/Divorced/Widowed	6 (9.7)	56 (90.3)	4.4 to 20.0
**Age at first sex**			
12 to 15	13 (7.7)	156 (92.3)	4.5 to 12.8
16 to 17	52 (9.7)	485 (90.3)	7.4 to 12.5
18 to 19	8 (6.0)	125 (94.0)	3.0 to 11.6
**Number of children alive**			
0	1 (3.2)	30 (96.8)	0.4 to 20.2
1	33 (12.0)	242 (88.0)	8.6 to 16.4
2	17 (7.2)	219 (92.8)	4.5 to 11.3
3–4	1 (2.0)	48 (98.0)	0.2 to 13.4
**Partner’s Education**			
None	1 (7.7)	12 (92.3)	1.0 to 41.2
Primary	17 (8.9)	173 (91.1)	5.6 to 13.9
Secondary	45 (10.6)	378 (89.4)	8.0 to 14.0
Tertiary	1 (5.0)	19 (95.0)	0.6 to 29.4
**Partner’s Occupation**			
Self-employed/Employed	22 (11.4)	171 (88.6)	7.6 to 16.7
Unemployed	24 (9.0)	243 (91.0)	6.1 to 13.1
Peasant farmer	7 (5.4)	122 (94.6)	2.6 to 11.0
Student	11 (19.3)	46 (80.7)	11.0 to 31.7

### Factors associated with modern contraceptive use among the study participants

Using logistic regression, variables which had a p-value less than 0.2 were considered significant at bivariate analysis. They were age (*p* = 0.153), marital status (*p* = 0.079), number of children alive (*p* = 0.081), partner’s age (*p* = 0.007), and partner’s occupation (*p* = 0.073). In addition, the variables that were plausible according to prior knowledge like education and ever been pregnant were added although their *p*-values were greater than 0.2. These variables were considered for the multivariate analysis. Statistical significance at multivariate analysis was considered at a p-value of 0.05. The significant variables at this level were being married (aOR = 0.11, 95% CI: 0.04 to 0.35, *p* < 0.001) or cohabiting (aOR = 0.43, 95% CI: 0.20 to 0.93, *p* = 0.032) and having an older partner (aOR = 0.93, 95% CI: 0.86 to 0.99, *p* = 0.046). The significant variables were further assessed for interaction between each other and for confounding with all the other independent variables that were significant at bivariate. However, there was neither interaction nor confounding. Therefore, marital status and partner’s age were the only variables associated with modern contraceptive use (Table 4).

**Table 4:** Factors associated with modern contraceptive use among refugee adolescents

Variable	Crude OR (95% CI)	*P*-Value	Adjusted OR (95% CI)	*P*-Value
**Age in years**				
Median (IQR): 19 (18–19)	0.83 (0.64 to 1.07)	0.153	0.75 (0.48 to 1.16)	0.194
**Marital status**				
Single	1.00		1.00	
Cohabiting	1.40 (0.79 to 2.46)	0.250	0.43 (0.20 to 0.93)	0.032
Married	0.43 (0.17 to 1.10)	0.079	0.11 (0.04 to 0.35)	< 0.001
Separated/Divorced/Widowed	1.21 (0.46 to 3.16)	0.702	0.29 (0.08 to 1.00)	0.050
**Number of children alive**				
Mean (SD): 1.5 (0.727)	0.70 (0.46 to 1.05)	0.081	0.83 (0.51 to 1.33)	0.431
**Partner’s age**				
Median (IQR): 26 (24–30)	0.91 (0.86–0.98)	0.007	0.93 (0.86 to 0.99)	0.046
**Partner’s Occupation**				
Self-employed/Employed	1.00			
Unemployed	0.77 (0.42 to 1.41)	0.396	0.82 (0.43 to 1.58)	0.553
Peasant farmer	0.45 (0.18 to 1.08)	0.073	0.49 (0.20 to 1.24)	0.134
Student	1.86 (0.84 to 4.11)	0.126		0.152

95% CI – 95% Confidence Interval

## Discussion

This study assessed the prevalence and factors associated with modern contraceptive use among female refugee adolescents in Palabek refugee settlement, Northern Uganda. The prevalence of modern contraceptive use was less than a tenth, and yet almost all (93%) did not want to become pregnant. Moreover, 70% of the respondents had ever been pregnant. The contraceptive prevalence rate in our study is much lower than that of the general population of married and unmarried sexually active female adolescents in Uganda, which was reported to be 25.2% [16]. Several studies from other refugee settings have reported similar findings of low modern contraceptive use among refugee adolescents [15, 22, 23]. For example, in a multi country study of refugee settings, current use of contraceptives was reported to be only 4% [22]. In addition, a study among refugees in Ghana reported current contraceptive use of 7.3% [23]. In previous studies, low prevalence of modern contraceptive use in refugee settings was attributed to inadequate knowledge on contraceptives, fear of side effects and partner prohibitions [18, 22, 24]. However, in our study knowledge of contraceptives was high and can therefore not explain the low prevalence of contraceptive use among adolescents in Palabek refugee settlement. The main reason for non-use was fear of side effects. The low prevalence could also be attributed to poor access to modern contraceptives since 70.2% of the participants could not access a contraceptive source within 10 min’ walk from their homes.

Over two thirds of the participants had ever been pregnant. This proportion is considerably higher than that in the general population of adolescents aged 15–19 years (28.8%) and 18–19 years (46.8%) in Uganda who have begun child bearing [16]. These findings highlight an urgent need for improved access to modern contraceptives in this population and setting.

The factors significantly associated with modern contraceptive use among the participants were marital status and partner’s age. Participants who were married and those who were cohabiting were 89 and 57% less likely to use modern contraceptives respectively than their counterparts who were single. In contrast, previous studies among non-refugees adolescents have shown that adolescents who were married were more likely to use modern contraceptives compared to those who were single [25, 26]. This inconsistency could have been brought about by the difference in setting and study population of the studies compared to ours. The association between modern contraceptive use and marital status observed in our study can be explained by the possibility that married adolescents could have been prohibited by their partners from using modern contraceptives. Partner prohibition emerged as the second commonest reason for contraceptive non-use in this study. This could explain why the adolescents who were married or cohabiting were less likely to use contraceptives than those who were single. Adolescents who were single had no regular partners to prohibit them from using modern contraceptives. Another possible explanation for this association is that adolescents who are married or cohabiting are more likely to desire to have children than those who are single. This is because many of them are expected by society to conceive after marriage [27]. Because of this, married adolescents or those who are cohabiting are less likely to opt for modern contraceptives.

The other factor that was significantly associated with modern contraceptive use was the partner’s age. Our results showed that modern contraceptive use decreased by 7% with every unit increase in the partner’s age. That is, adolescents with older partners were less likely to use modern contraceptives than those with partners their age. In contrast, a previous study among married women aged 15 to 49 years in Nigeria showed no association between partner’s age and modern contraceptive use [28]. This discrepancy could be explained by differences in age inclusion and supports our postulation that adolescents have lower negotiating power compared with adult women, especially surrounding matters concerning their sexual and reproductive health. This is as explained by the gender and power theory [29]. The fact that gender and power imbalances deepen with increasing age disparities within couples has been confirmed in previous studies [30, 31].

Some of the strengths of this study are: standardised approaches like pre-testing of questionnaires, translation and back translation of questionnaires to ensure that meaning is not altered, were used to carry out this study. This helped to minimise misclassification bias, and increases the ability to repeat and replicate the findings. Furthermore, a large sample size was used and this gave the study a high power enough to answer its research questions.

However, this study has some limitations. First, the sample was not representative because a non-random sampling technique (consecutive sampling) was used. However, this was minimised by collecting the sample from all sections of the refugee settlement. Secondly, this study used questionnaires to obtain sensitive information on sexual and reproductive history of the participants. These questions are subject to social desirability bias where participants are likely to give responses that are socially acceptable instead of the true responses. This bias was minimised by conducting interviews in calm and friendly environments. Finally, this was a cross-sectional study so causal relationships could not be established.

## Conclusion

In conclusion, less than a tenth of the participants were using modern contraceptives, and this leaves them vulnerable to adolescent pregnancies and their associated complications. The most at risk groups were adolescents who were either married or cohabiting and those with much older partners. The main reasons for not using modern contraceptives were fear of side effects, partner prohibition and lack of knowledge.

Our study shows that there is an urgent need for measures to improve adolescents’ access to high-quality sexual and reproductive healthcare in refugee settings. Future interventions should empower adolescents in refugee settings to make decisions about their own health including initiating a contraceptive method, address their fear of side effects and knowledge gaps, and ensure better contraceptive counselling techniques so as to improve use of modern contraceptives.

## Data Availability

The datasets used and/or analysed during the current study are available from the corresponding author on reasonable request.

## Abbreviations

IUDs: Intra-uterine devices
EFC: Evangelical Free Church
AIC: African Initiated Church
CI: Confidence Interval
OR: Odds Ratio
SD: Standard Deviation
